# A Comparison of Transcriptional Patterns and Mycological Phenotypes following Infection of *Fusarium graminearum* by Four Mycoviruses

**DOI:** 10.1371/journal.pone.0100989

**Published:** 2014-06-25

**Authors:** Kyung-Mi Lee, Won Kyong Cho, Jisuk Yu, Moonil Son, Hoseong Choi, Kyunghun Min, Yin-Won Lee, Kook-Hyung Kim

**Affiliations:** Department of Agricultural Biotechnology, Center for Fungal Pathogenesis, and Research Institute of Agriculture and Life Sciences, College of Agriculture and Life Sciences, Seoul National University, Seoul, Republic of Korea; University of Wisconsin – Madison, United States of America

## Abstract

Many fungi-infecting viruses, which are termed mycoviruses, have been identified, and most do not cause any visible symptoms. Some mycoviruses, however, can attenuate the virulence of the infected fungi, a phenomenon referred to as hypovirulence. To study fungus responses to virus infection, we established a model system composed of *Fusarium graminearum* and four mycoviruses including FgV1 (Fusarium graminearum virus 1), FgV2, FgV3, and FgV4. FgV1 and FgV2 infections caused several phenotypic alterations in *F. graminearum* including abnormal colony morphology, defects in perithecium development, and reductions in growth rate, conidiation, and virulence. In contrast, FgV3 and FgV4 infections did not cause any phenotypic change. An RNA-Seq-based analysis of the host transcriptome identified four unique *Fusarium* transcriptomes, one for each of the four mycoviruses. Unexpectedly, the fungal host transcriptome was more affected by FgV1 and FgV4 infections than by FgV2 and FgV3 infections. Gene ontology (GO) enrichment analysis revealed that FgV1 and FgV3 infections resulted in down-regulation of host genes required for cellular transport systems. FgV4 infection reduced the expression of genes involved in RNA processing and ribosome assembly. We also found 12 genes that were differentially expressed in response to all four mycovirus infections. Unfortunately, functions of most of these genes are still unknown. Taken together, our analysis provides further detailed insights into the interactions between mycoviruses and *F. graminearum*.

## Introduction

Viruses that infect fungi consist of single-stranded or double-stranded (ds) RNAs and are referred to as mycoviruses. Many mycoviruses with dsRNA genomes have been identified and divided into five families [1]. Most mycoviruses do not cause visible symptoms in the host fungus, but some can reduce the virulence of plant-pathogenic fungi in a phenomenon that is termed hypovirulence [1]. Hypovirulence caused by mycovirus infections can suppress plant-pathogenic fungi and thereby reduce the need for fungicide applications. With the development of hyphal or protoplast fusion techniques, hypovirulent mycoviruses could be transmitted to other fungal pathogens [2], [3].

The current study concerns mycoviruses of *Fusarium graminearum.* Members of the *Fusarium graminearum* (Fg) species complex are important plant pathogens that damage wheat, barley, maize, and other cereal crops by reducing yield and by producing mycotoxins [4]. Now that the complete genome sequence for *F. graminearum* strain PH-1 has been published [5], it has become possible to study the population structure, multi-omics, gene function, sexual development, mycotoxins, and pathogenicity of *F. graminearum* at the molecular level [6], [7].

Several mycoviruses have been identified in *Fusarium* species [8], and the complete genome sequences for at least seven Fusarium mycoviruses are currently available [9]–[13]. Our laboratory has reported on four mycoviruses identified from the *F. graminearum* species complex isolated from diseased maize and barley in Korea [14]. These mycoviruses were designated as Fusarium graminearum virus 1-DK21 (FgV1-DK21; hereafter referred to as FgV1) and Fusarium graminearum viruses 2, 3, and 4 (hereafter referred to as FgV2, FgV3, and FgV4, respectively). The four mycoviruses consist of one to five different segments of dsRNA ranging in size from approximately 1.7 to 9.3 kb [10], [13], [15]. FgV1 was the first mycovirus of *Fusarium* species found to reduce fungal virulence and growth rate, to change colony morphology, and to increase pigmentation [10]. The other three mycoviruses (FgV2, FgV3, and FgV4) have not yet been characterized in detail. Moreover, the newly identified Fusarium graminearum virus-china9 (FgV-ch9), which is closely related to FgV2 based on genome organization and sequence identify, caused hypovirulence and related phenotypes in *F. graminearum* [9], [16].

A limited number of studies have demonstrated transcriptional or translational changes in the fungal host following mycovirus infection [17]. For instance, 80 *Cryphonectria parasitica* genes involved in viral RNA replication and cellular defense have been identified using a cDNA microarray representing 2,200 genes [17]. A genome-wide transcriptome analysis of *F. graminearum* infected with FgV1 was recently published; the study used a 3′-tiling microarray and revealed that genes affecting transcription and translation machinery were up-regulated while those affecting metabolism and transport systems were down-regulated [18]. A two-dimensional gel electrophoresis (2-DE)-based proteomic analysis identified several differentially expressed *F. graminearum* proteins upon FgV1 infection, and these included proteins associated with differentiation, antioxidant activities, and glycolysis [19].

The goal of the current study was to identify host genes involved in the interaction between mycovirus and fungus host. We established a model system with *F. graminearum* as the host and with four mycoviruses that infect members of the *F. graminearum* species complex. By using the *F. graminearum* genome sequence and next generation sequencing technology, we conducted a comprehensive genome-wide transcriptome analysis to identify potential genes involved in the many biological processes associated with the mycovirus–host interaction.

## Results

### Phenotypes of *F. graminearum* caused by four mycoviruses

To investigate phenotypes of *F. graminearum* host caused by different mycoviruses, we first used protoplast fusion to generate four *F. graminearum* strains infected with *Fusarium* mycoviruses, such as FgV1, FgV2, FgV3, or FgV4. The previously identified *Fusarium* isolates DK21 (FgV1-infected), 98-8-60 (FgV2-infected), and DK3 (co-infected with FgV3 and FgV4) were used as donors, and the wild-type (WT) *F. graminearum* strain PH-1 was used as the recipient for protoplast fusion (Table 1). The protoplast fusants were selected based on enzyme treatment, RT-PCR analysis, Southern blot hybridization, AFLP, and sequence analysis using two genes, one encoding TEF-1α (translation elongation factor 1α) and the other encoding histone H3 (Figures S1 and S2 in File S1). The generated *F. graminearum* strains were designated PH-1/FgV1, PH-1/FgV2, PH-1/FgV3, and PH-1/FgV4.

**Table 1:** *Fusarium* strains used in this study.

Strain	Characteristics	References
DK21	*Fusarium boothii* (lineage 3) infected with FgV1-DK21	
98-8-60	*F. asiaticum* (lineage 6) infected with FgV2	
DK3	*F. graminearum* (lineage 7) infected with FgV3 and FgV4	
PH-1	Wild-type *F. graminearum* (lineage 7)	
PH-1/FgV1	PH-1 infected with FgV1-DK21	This study
PH-1/FgV2	PH-1 infected with FgV2	This study
PH-1/FgV3	PH-1 infected with FgV3	This study
PH-1/FgV4	PH-1 infected with FgV4	This study

**Figure 1:**
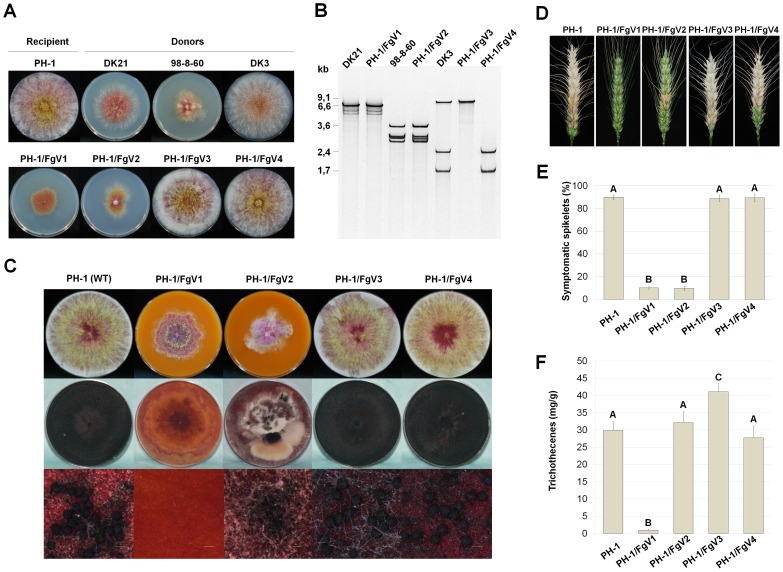
Effects of *Fusarium* mycovirus infections on *F. graminearum* strain PH-1. PH-1/FgV1, PH-1/FgV2, PH-1/FgV3, and PH-1/FgV4 indicate the PH-1 strain infected with FgV1, FgV2, FgV3, and FgV4, respectively. (A) Colony morphology of *Fusarium* strains used in this study. PH-1 strains infected with FgV1–FgV4 were obtained by protoplast fusion. DK21 and 98-8-60 are FgV1 and FgV2-infected strains, respectively, whereas DK3 is an FgV3/4-coinfected strain. Cultures were photographed after 5 days on PDA. (B) Viral dsRNAs were extracted from mycelia using CF-11 cellulose chromatography. The presence of dsRNAs was confirmed by 5% polyacrylamide gel; the gels were stained with EtBr and visualized with a UV-transilluminator. (C) Sexual development of each strain. The strains were incubated on carrot agar medium for 7 days (upper row) and then treated with a Tween-60 solution to induce sexual reproduction (middle row). Cultures were examined for perithecia after 7 days under UV light. Scale bar  = 200 µm. (D) Fusarium head blight symptoms caused by PH-1 (WT) or mycovirus-infected strains PH-1/FgV1–4. (E) Disease severity was evaluated 14 days after inoculation. (F) Trichothecene production. Conidial suspensions were grown in minimal medium containing 5 mM agmatine. After 7 days, trichothecenes in the culture filtrates were quantified by GC-MS. For (E) and (F), data were analyzed by the General Lineal Model (GLM) using IBM SPSS statistics 20 for Windows software. Error bars indicate standard deviations. Different letters above the bars indicate significant differences at *p* = 0.05.

The colony morphologies of all four mycovirus-infected strains (recipients) were comparable to those of the mycovirus-infected donor strains (Figure 1A). Relative to the colony diameter of the virus-free PH-1 strain, the colony diameter was reduced for PH-1/FgV1 and PH-1/FgV2 but not for PH-1/FgV3 or PH-1/FgV4 (Figure 1A, Table 2). In addition to a reduced growth rate, colonies of PH-1/FgV1 and PH-1/FgV2 also had significant morphological alterations including increased pigmentation and irregular margins (Figure 1A). The colonies of PH-1/FgV3 and PH-1/FgV4, in contrast, appeared similar to those of the virus-free PH-1 strain. Electrophoretic analysis indicated that dsRNAs of FgV1 to FgV4 purified from subcultured protoplast fusants had the same mobility on 5% polyacrylamide gels as those purified from donor strains, DK21, 98-8-60, and DK3 (Figure 1B).

**Table 2:** Phenotypic characteristics of *F. graminearum* PH-1 when infected by FgV1, 2, 3, or 4.

Strain	Colony diameter^a^ (mm)	Conidia produced per culture^b^ (10^5^/ml)	Conidium morphology^c^	
			Length (µm)	Width (µm)
PH-1	43.25^A^±0.85	2.92^A^±0.93	41.3^A^±1.35	5.27^A^±0.28
PH-1/FgV1	25.04^B^±5.00	3.55^A^±0.55	26.7^B^±6.70	5.64^B^±0.64
PH-1/FgV2	23.08^C^±3.08	0.43^B^±0.44	39.9^A^±9.98	5.33^A^±0.34
PH-1/FgV3	43.17^A^±3.17	3.50^A^±0.51	41.5^A^±1.57	5.31^A^±0.32
PH-1/FgV4	44.71^A^±4.71	2.92^A^±0.92	39.3^A^±9.31	5.26^A^±0.26

Within columns, means with different letters are significantly different according to Duncan's multiple range test (*p* = 0.05).

a Colonies were measured after 5 days on PDA.

b Conidia were counted in 5-day-old CMC cultures.

c Conidia were harvested from CMC cultures, and 100 were observed per strain with a light microscope.

In an assay for sexual development, both PH-1/FgV3 and PH-1/FgV4 produced normal perithecia (Figure 1C). In contrast, PH-1/FgV1 failed to produce perithecia or perithecial initials, and PH-1/FgV2 formed a few immature perithecia but failed to form mature perithecia. The perithecia of PH-1/FgV3 and PH-1/FgV4 FgV1 contained normal asci with normal ascospores but the immature perithecia of PH-1/FgV2 contained a reduced number of asci rosettes with abnormal ascospores (Figure S3 in File S1).

In an assay for the conidiation and conidial morphology, PH-1/FgV2 produced a reduced number of conidia while PH-1/FgV1 produced shorter and wider conidia than the virus-free PH-1 strain in CMC culture (Table 2 and Figure S4 in File S1). This swollen and two-celled shape is similar to microconidia formed by other *Fusarium* species. This observation indicated that conidial formation and/or maturation process might be interrupted by FgV1 infection. We also examined the vertical transmission of mycoviruses via conidia through three generations (Table 3). FgV1 was transmitted to all or almost all conidia in all three generations, and the transmission of FgV2 increased with each generation. Transmission of FgV4, in contrast, tended to decrease and that of FgV3 was inconsistent.

**Table 3:** Vertical transmission of *Fusarium* mycoviruses.

Conidial generation	Vertical transmission rate (%)			
	FgV1	FgV2	FgV3	FgV4
**1^st^**	93.3 [28/30]	10.0 [3/30]	56.7 [17/30]	100 [30/30]
**2^nd^**	93.3 [28/30]	83.3 [25/30]	46.7 [14/30]	40.0 [12/30]
**3^rd^**	100 [30/30]	100 [30/30]	76.7 [23/30]	36.7 [11/30]

Vertical transmission was measured as the percentage of FgVs-positive isolates among the total number of single-conidium isolates. The presence of viral dsRNA was determined by RT-PCR analysis. The mycelial plugs obtained from virus-positive isolates were inoculated into CMC liquid medium for the next conidial generation. Numbers in squared brackets indicate the number of virus-positive isolates/total number of single-conidium isolates.

In a virulence assay, the virus-free stain and all four mycovirus-infected strains caused at least some degree of Fusarium head blight 2 weeks after inoculation (Figure 1D). Based on symptoms, virulence was greatly reduced in PH-1/FgV1 and PH-1/FgV2 but not in PH-1/FgV3 or PH-1/FgV4 (Figures 1D and 1E).

We also compared mycotoxin production for each fungal strain. The level of trichothecenes produced was highest for PH-1/FgV3; intermediate for PH-1/FgV2, PH-1/FgV4, and virus-free PH-1; and lowest for PH-1/FgV1 (Figure 1F).

### Genome-wide analysis of host gene expression using RNA-Seq

**Figure 2:**
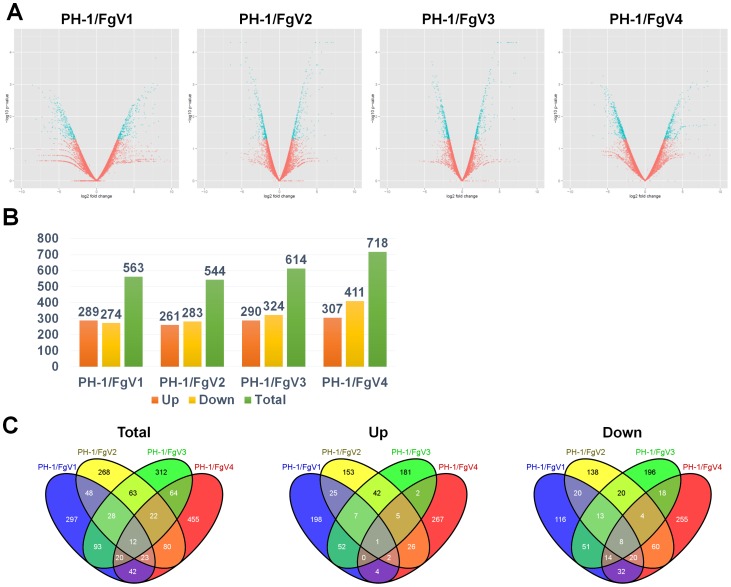
*F. graminearum* genes that were differentially expressed in response to four mycoviruses and that were identified by RNA-Seq. (A) Volcano plot of RNA-Seq data using log_2_fold change and log_10_p-value. X and Y axes represent log_2_-converted fold change and log_10_-converted p-value. (B) The number of DEGs. Orange, yellow, and green colors indicate number of DEGs for up-regulated, down-regulated, and total of up- and down-regulated genes, respectively. (C) Venn diagrams illustrating the number of genes that were differentially expressed in subsets of the four virus-infected strains. Total, down, and up indicate total numbers of DEGs, the numbers of up-regulated DEGs, and the numbers of down-regulated DEGs, respectively.

Having determined that different mycoviruses caused different phenotypes in *F. graminearum*, we next examined the effects of the mycoviruses on host gene expression, and we attempted to identify genes linked to the phenotypic changes and especially to hypovirulence. We performed a genome-wide transcriptome analysis using RNA-Seq. The mycelia of the four mycovirus-infected strains and the virus-free strain of *F. graminearum* were harvested after 5 days in shake culture. Total RNA was isolated, and five cDNA libraries were constructed as described in the Materials and Methods. The five libraries were sequenced by the Illuminia HiSeq 2000. The obtained reads were mapped on the reference sequences for *F. graminearum* PH-1, which contains a total of 13,322 genes with a length of 17,842,161 bp. We used Tophat and Cufflinks to align reads on the reference genomes and to assemble the reads into transcripts, respectively. Finally, Cuffdiff implemented in Cufflinks was used to identify differentially expressed genes (DEGs) via several statistical analyses (Table S1 in File S2). As a result, FPKM, fragments per kilobase of transcript per million fragments mapped, were calculated for expression values. To identify DEGs, we used a threshold of a 2-fold change in expression relative to the virus-free sample and p-value less than 0.05. Based on obtained fold changes and p-values, we generated volcano plots to display the gene expression pattern in each sample (Figure 2A). The volcano plots indicate that the host transcriptome was affected more by FgV1 and FgV4 than by FgV2 or FgV3. The number of up-regulated genes ranged from 261 genes (1.95%) to 307 genes (2.3%) while the number of down-regulated genes ranged from 274 genes (2.05%) to 411 genes (3.08%) (Figure 2B). The number of DEGs was the highest in PH-1/FgV4 (718 genes) and the lowest in PH-1/FgV2 (544 genes).

We next compared DEGs to determine the number of virus-specific or commonly expressed genes in the four samples obtained from mycovirus-infected mycelia (Figure 2C). Only 12 genes were commonly identified in the four samples, and the number of specifically expressed genes in each virus-infected sample was 297 for PH1/FgV1, 268 for PH1/FgV2, 312 for PH1/FgV3, and 455 for PH1/FgV4 (Figure 2C). All redundant DEGs in the four samples were combined, and a total of 1,827 non-redundant DEGs, representing 13.71% of the total genes, were determined to be differentially expressed. Of them, 965 genes (7.24%) were down-regulated, and 965 genes (7.24%) were up-regulated by mycovirus infection. We further divided the DEGs into those that were down-regulated in all four samples or up-regulated in all four samples. Only one gene (FGSG_06969) related to F-box protein Fbl2 were up-regulated in all four samples, and eight genes were down-regulated in all four samples (Table 4).

**Table 4:** The 12 DEGs commonly identified in response to four different mycovirus infections by RNA-Seq.

Locus	Description	PH-1/FgV1		PH-1/FgV2		PH-1/FgV3		PH-1/FgV4	
		log2(FC)	p_value	log2(FC)	p_value	log2(FC)	p_value	log2(FC)	p_value
FGSG_00878	hypothetical protein	−4.91648	0.0052	−2.01029	0.02615	−2.05761	0.02695	−3.12042	0.0215
FGSG_01695	hypothetical protein	−3.72934	0.0245	−1.87601	0.04125	−2.06414	0.0312	−2.91781	0.0393
FGSG_02578	hypothetical protein	3.56003	0.0435	−2.44035	0.0067	2.12449	0.02755	−2.90501	0.02865
FGSG_04917	hypothetical protein	−4.25978	0.0107	−2.01776	0.0245	−2.43134	0.01045	−2.63378	0.04985
FGSG_05471	similar to aspartic proteinase OPSB	3.31593	0.0368	−1.7438	0.04855	2.12627	0.01645	−3.05869	0.0279
FGSG_05695	CAT1 catalase	−5.25633	0.0095	−2.88885	0.00295	−1.93356	0.0275	−4.26937	0.00365
FGSG_06969	F-box protein Fbl2	4.39156	0.01665	3.49683	0.0023	3.14555	0.0029	2.89172	0.04295
FGSG_07582	similar to monosaccharide transporter	−5.39624	0.00275	−1.82755	0.04485	−2.73038	0.00585	−3.94659	0.00805
FGSG_07587	similar to hexose carrier protein	−3.62644	0.0219	1.83566	0.03675	−1.99288	0.023	−3.12381	0.02275
FGSG_09059	hypothetical protein	−4.2155	0.01935	−1.83773	0.0324	−2.00734	0.0229	−4.27429	0.005
FGSG_10425	hypothetical protein	−4.1896	0.0099	−1.77707	0.03755	−1.78205	0.04225	−3.28925	0.0148
FGSG_10926	hypothetical protein	−5.07082	0.0153	−2.78263	0.00405	−2.29	0.00965	−3.87388	0.0081

### Functional distribution of host genes that were differentially expressed in response to infection by four mycovirus

We selected the top 20 DEGs in each sample that exhibited the greatest change in expression (Table S2 in File S2). Because many fungal genes are unknown, only a limited number of genes were functionally annotated. In PH1/FgV1, the most strongly down-regulated gene was the gene encoding conserved hypothetical protein (FGSG_07822), and the most strongly up-regulated gene was conserved hypothetical protein (FGSG_07804). In PH1/FgV1, several genes encoding alkaline proteinase, cytochrome P450 phenylacetate hydroxylase, and glutathione-dependent formaldehyde dehydrogenase were strongly down-regulated, but many genes encoding hypothetical proteins were up-regulated. In both PH1/FgV2 and PH1/FgV3, the levanbiose-producing levanase gene (FGSG_06451) was down-regulated while four genes encoding hypothetical proteins were up-regulated. In PH1/FgV4, genes related to nitrite reductase, acetyltransferase, and flavohemoglobin were down-regulated while a gene for fruit body lectin was up-regulated.

### Gene ontology enrichment analysis of DEGs

To obtain insight into essential gene functions regulated by mycovirus infection, we conducted gene ontology (GO) enrichment analysis. The DEGs in each sample were divided into up-regulated and down-regulated genes. A total of eight gene lists were subjected to GO enrichment analysis, and several enriched GO terms were identified in only down-regulated gene lists for PH-1/FgV1, PH-1/FgV3, and PH-1/FgV4 (Table S3 in File S2). Only four and seven GO terms were enriched in down-regulated genes for PH1/FgV1 and PH1/FgV3, respectively. In contrast, 58 GO terms were enriched in down-regulated genes for PH1/FgV4.

In the group of PH-1 genes that were down-regulated by FgV1 infection, genes involved in transporting activity such as potassium and sodium-transporting ATPase activity were identified (Table S3 in File S2). Similarly, genes associated with transporting activity such as monosaccharide, carbohydrate, polyol, and hexose transport, were strongly down-regulated by FgV3 infection (Table S3 in File S2). FgV4 infection reduced the expression of genes involved in RNA processing (GO:0006396) (Table S3 in File S2). These genes are associated with RNA 5′-end processing (GO:0000966), ncRNA processing (GO:0034470), and rRNA metabolic process (GO:0016072) (Figure S5 in File S1). In particular, these genes function in processing, maturation, and endonucleolytic cleavage of ribosomal RNAs. In addition, genes associated with ribosome biogenesis (GO:0042254) including ribosome assembly (GO:0042255) were strongly down-regulated (Table S3 in File S2). According to the cellular component, genes encoding subunits of nucleus (GO:0005634) and preribosome (GO:0030684) were strongly down-regulated by FgV4 infection (Figure S6 in File S1).

### Effect of mycovirus infection on expression of transcription factors

**Figure 3:**
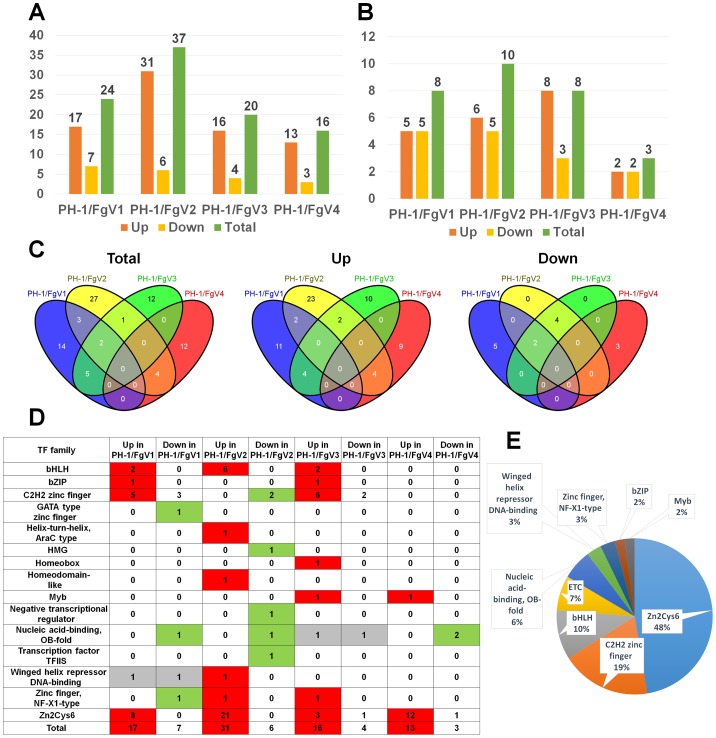
*Fusarium graminearum* transcription factors that were differentially expressed in response to mycovirus infection. The number of TFs (A) and the number of TF families (B) that were differentially expressed in response to infection by the four mycoviruses. (C) Venn diagrams illustrating the numbers of TFs that were differentially expressed in subsets of the four virus-infected strains. Total, up, and down indicate the total numbers of DEGs, the numbers of up-regulated DEGs, and the numbers of down-regulated DEGs, respectively. (D) The number of differentially expressed TFs belonging to 15 representative TF families. Red indicates that the number of differentially up-regulated genes was greater than the number of down-regulated genes; green indicates the opposite. (F) The percentage of TF families in all identified differentially expressed TFs.

Transcription factors (TFs) could play important roles in the transcriptional regulation of host genes by mycovirus infection. A previous study reported that the genome of *F. graminearum* contains at least 659 TFs belonging to 44 families [20] (Table S4 in File S2). To examine changes in the expression of TFs, we identified differentially expressed TFs. The number of differentially expressed TF genes ranged from 16 to 37 genes (Figure 3A). A total of 37 and 16 TFs were differentially expressed in response to FgV2 and FgV4 infection, respectively. In particular, the number of up-regulated genes was five-times greater than the number of down-regulated genes in the FgV2-infected sample. We also examined the distribution of differentially expressed TF families in each sample (Figure 3B). From three to eight TF families were differentially expressed by different mycovirus infection. In the FgV1-infected sample, five TF families were up-regulated while five TF families were down-regulated. We next compared the number of differentially expressed TFs in the four samples by family (Figure 3C). Of 80 differentially expressed TFs, including 14 that were down-regulated and 65 that were up-regulated, were differentially expressed. Although almost 13.35% of the TFs were differentially expressed by at least one mycovirus infection, no TF was commonly identified in all four FgV-infected samples (Figure 3C). Up-regulated TFs were more numerous than down-regulated TFs in all four samples (Figure 3D). We further examined the portion of TF families which were enriched in DEGs. The Zn2Cys6 family (48%) was the dominant TF family followed by C2H2 zinc finger (19%) and bHLH (10%) TF families (Figure 3E). At least 15 TF families were differentially expressed in response to different mycovirus infection.

### Expression of genes involved in post-transcriptional gene silencing

We examined the expression of genes involved in post-transcriptional gene silencing (PTGS). The *Fusarium* genome contains two argonaute-like genes (*ago*), five dicer-like genes (*dicer*), and five RNA-directed RNA polymerase (*rdr*) genes (Table S5 in File S2). Expression of FGSG_08752 (*ago*) was significantly up-regulated by FgV2 and FgV3 infections. Another *ago* gene (FGSG_00348) did not show reliable expression data. Of four *dicer* genes, expression of FGSG_04408 showed strong up-regulation by FgV2 and FgV3 infection. In general, most *rdr* genes were frequently over-expressed in response to different mycovirus infection. In particular, FGSG_09076, FGSG_04619, and FGSG_01582 were strongly up-regulated by FgV2 and FgV3 infections. Of the five *rdr* genes, expression of FGSG_06504 was strongly up-regulated by FgV4 infection.

### Validation of RNA-Seq results by real time RT-PCR

**Figure 4:**
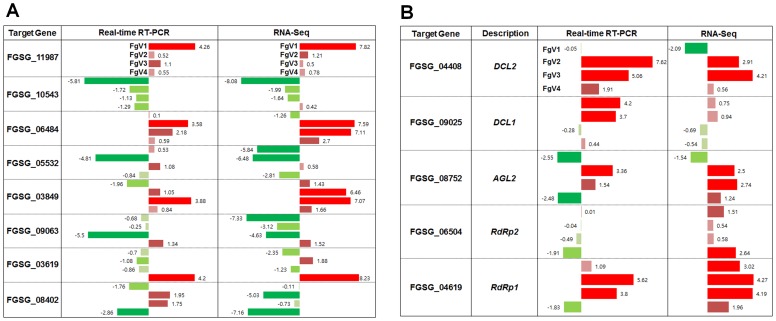
Validation of RNA-Seq data by real-time RT-PCR. The expression of eight selected genes (A) and five genes involved in RNAi silencing (B) was examined by real-time RT-PCR. Up-regulation and down-regulation of selected genes were indicated by red and green bars with corresponding fold changes, respectively.

To confirm RNA-Seq results, we selected a total of 13 genes and prepared total RNAs from different biological samples. Two genes encoding cyclophilin 1 (FGSG_07439) and elongation factor 1α (FGSG_08811) were used as reference genes to normalized real time RT-PCR. We performed real time RT-PCR at least three times with gene specific primers (Table S6 in File S2). As shown in Figure 4, the results of RNA-Seq were highly consistent with those of real-time RT-PCR in general. For example, the expression of FGSG_11987 was strongly up-regulated by all four mycoviruses which were confirmed by both RNA-Seq and real-time RT-PCR approaches (Figure 4A). By contrast, some genes such as FGSG_03619 and FGSG_08402 showed difference between RNA-Seq and real time RT-PCR results (Figure 4A). In addition, we performed real-time RT-PCR for five genes involved in RNAi silencing (Figure 4B). For instance, expressions of *dcl2* were strongly up-regulated by three mycoviruses except FgV1.

### Comparative analysis of RNA-Seq vs. microarray data

Previously, we used the microarray system to analyze genome-wide gene expression of *F. graminearum* in response to FgV1 infection [18]. The microarray data from the latter study and the RNA-Seq data from the current study were compared. Specifically, we compared three data sets containing 5,567 DEGs identified by RNA-Seq at 120 hours post-infection (hpi), 1,109 DEGs identified by microarray at 36 hpi, and 1,050 DEGs identified by microarray at 120 hpi (Figure S7 in File S1). Only 41 DEGs were commonly identified in the three data sets, and 140 DEGs were commonly identified by RNA-Seq and microarray at 120 hpi.

## Discussion

In this study, we established a unified model system to study mycovirus and fungal host interaction. Our system consists of four mycoviruses of *Fusarium* species and a fungal host (the PH-1 strain of *F. graminearum*) for which the whole genome sequence is available. Protoplast fusion was used to infect *F. graminearum* PH-1 with each mycovirus. The colony morphologies of all four mycovirus-infected strains (recipients) were similar to the morphologies of the donor strains. Although the *F. graminearum* donor strain DK3 was co-infected with FgV3 and FgV4, our protoplast-based fusion approach successfully generated a strain containing only a single mycovirus. This result indicates that our methods could be used to isolate a specific virus and to characterize the reaction of the target host to infection by that virus.

Although the four mycoviruses used in this study belonged to four different families, they could also be divided into two groups based on virulence: FgV1 and FgV2 were hypovirulent, and FgV3 and FgV4 were non-hypovirulent. When infected by the two hypovirulent mycoviruses, *F. graminearum* PH-1 exhibited similar phenotypes, such as a reduction in growth rate, sexual development, and virulence; an increase in pigmentation; and irregular margins of colonies on PDA medium. In contrast, *F. graminearum* PH-1 phenotypes were not changed when PH-1 was infected by either of the two non-hypovirulent mycoviruses. These data indicate that not all mycoviruses cause similar visible symptoms in the infected fungal host. It is highly likely, however, that non-virulent mycoviruses could become virulent in response to unknown stimuli or in other fungal hosts.

We expected that FgV1 and FgV2 infections would generate similar responses in *F. graminearum* PH-1 but that was not always the case. Thus, PH-1/FgV1 exhibited defects in growth, toxin production, conidial size, and sexual development but not in conidiation, whereas PH-1/FgV2 exhibited defects in growth, conidiation, and sexual development but not in toxin production or conidial size. In addition, the transcript expression profile of PH-1/FgV1 did not match that of PH-1/FgV2. These results indicate that there is no direct relation between transcriptional profiling and associated gene functions controlling toxin production, asexual development, and sexual development between hypovirulent FgV1 and FgV2 in their fungal host.

The asexual transmission rate of the mycoviruses was correlated with hypovirulence in that transmission of the hypovirulent mycoviruses was high (FgV1) or increased with consecutive conidial generations (FgV2) while transmission of the non-hypovirulent mycoviruses declined (FgV4) or did not show a consistent patter with conidial generation (FgV3). Their high transmissibility suggests that the hypovirulent mycoviruses (FgV1 and FgV2) are better adapted than the non-hypovirulent mycoviruses (FgV3 and FgV4) to the host *F. graminearum* PH-1.

An RNA-Seq-based genome-wide expression analysis revealed four unique *Fusarium* transcriptomes regulated by four phylogenetically different mycoviruses. RNA-Seq showed that approximately 90% of genes were expressed and that the number of DEGs was less than that of microarray analysis. The different results obtained with microarray and RNA-Seq approaches can be explained by several technological differences of RNA-Seq and statistical analysis [21], [22]. Moreover, it was surprising that the fold changes of *Fusarium* genes that were differentially expressed in response to each mycovirus was much higher than the percentage expressed by plants in response to virus infection, indicating that a large number of fungal genes are regulated by mycoviruses.

Before conducting the expression analysis, we assumed that the two hypovirulent mycoviruses would have a stronger effect than the non-hypovirulent mycoviruses on the *Fusarium* transcriptome. As expected, we observed more transcriptional changes in response to the hypovirulent FgV1 than to the non-hypovirulent FgV3. The expression profiles caused by FgV2 and FgV4 infections, however, were unexpected. Transcriptional change caused by non-hypovirulent FgV3 was comparable to that caused by hypovirulent FgV2 while transcriptional change caused by non-hypovirulent FgV4 was greater than that caused by hypovirulent FgV2. These data indicate that the phenotypes observed for mycovirus-infected fungal hosts are not always correlated with the number of DEGs. The number of DEGs induced by infection was similar for the non-hypovirulent FgV4 and the hypovirulent FgV1, indicating that even non-hypovirulent mycoviruses can actively participate in host gene expression.

The enriched GO terms for genes that were down-regulated by FgV1 infection are highly associated with transporting activity, such as potassium and sodium-transporting ATPase activity. These data indicate that FgV1 strongly suppresses the host cellular transporting system. This result is consistent with the previous microarray analysis reporting down-regulation of genes associated with transporting system localizing to transmembrane, although the two approaches, RNA-Seq and microarray, identified different sets of DEGs.

Although the PH-1/FgV2 strain displayed abnormal phenotypes and hypovirulent characteristics, none of enriched GO term was identified. Most other DEGs were hypothetical genes and thus could not be assigned to known GO terms. These results indicate that only a limited number of *Fusarium* genes have been assigned to known GO terms and that most *Fusarium* genes are not orthologous to known genes in other eukaryotic organisms. Thus, it is quite difficult to find genes or gene functions associated with infection by FgV2. In contrast to FgV1, FgV4 infection down-regulated the expression of genes involved in RNA processing and ribosomal assembly. These genes mostly encode proteins that are components of the nucleolus and ribosome. Unexpectedly, we found it difficult to find correlation between gene functions and each mycovirus infection because each mycovirus regulates the expression of a totally different set of host genes. Although a large number of DEGs were virus-specific, 12 DEGs were common to PH-1/FgV1, PH-1/FgV2, PH-1/FgV3, and PH-1/FgV4, suggesting that they could be involved in stress response. Except few genes, functions of most genes are unknown.

We found that a majority of *Fusarium* TFs were differentially expressed by each mycovirus suggesting that fungal TFs might have important roles in the response to mycovirus infection. Of known TF families, the Zn2Cys6 TF family is fungal-specific and dominant [23]. Interestingly, the number of up-regulated TFs was always higher than the number of down-regulated TFs, suggesting that mycoviruses might utilize host TFs for their replication. However, the *F. graminearum* TF sets whose expression was significantly affected by infection differed greatly among the four mycoviruses, suggesting that the effect of infection on TF expression is mycovirus-specific.

We examined the effect of the four mycoviruses on the expression of 12 *Fusarium* genes that are responsible for PTGS. Among them, an *rdr* gene (FGSG_04619) was strongly up-regulated by all mycoviruses, suggesting that it might have an important role in PTGS. The effect of mycovirus infection on the expression of other PTGS-related genes seems to be very virus-specific. The biological functions of the RNA silencing pathway have been characterized in the model fungus *Neurospora crassa* [24]. A previous study showed that expression of genes involved in dsRNA-triggered gene silencing is strongly induced in response to viral infection [25]. For instance, both CHV1 and *Aspergillus* virus 341 are the targets of the RNA silencing machinery [26], [27]. Whereas CHV1 infections cause growth retardation in the absence of DCL2 or AGL2, *Aspergillus* virus 341 infections do not change the phenotype of *A. nidulans* strains lacking Dicer, Argonaute, and two RDRs [28]. The major RNAi components in the *F. graminearum* responsible for transcriptional regulation and antiviral mechanism remain unclear. Thus, it might be useful to study their roles in the response of *F. graminearum* to mycovirus infection.

In summary, we have described a model system for the study of mycovirus–host interactions. This system involves *F. graminearum* and four phylogenetically different mycoviruses. Phenotypic analysis revealed hypovirulent-related characteristics when *F. graminearum* PH-1 was infected by FgV1 or FgV2. Furthermore, RNA-Seq-based genome-wide gene expression analysis elucidated four unique *Fusarium* transcriptomes (one for each combination of four mycoviruses and the host *F. graminearum* PH-1). Our results also provide evidence that changes in the host transcriptome caused by different mycoviruses are not always correlated with observed host phenotypes.

## Materials and Methods

### Fungal strains and culture conditions

All strains used in this study were stored in 15% (v/v) glycerol at −80°C and were reactivated on Difco potato dextrose agar (PDA) (BD, New Jersey, U.S.A.). Fungal strains used for extractions of total RNA and genomic DNA were grown in 50 ml of liquid complete medium (CM) at 25°C at 150 rpm for 5 days. Mycelia were harvested by filtration through Whatman 3MM filter paper (GE Healthcare, Uppsala, Sweden), washed with distilled water, pressed between paper towels to remove the excess water, and stored at −80°C.

### Protoplast fusion

Protoplast fusion was performed according to the previous study [2]. Protoplast fusants were selected with hygromycin B at a final concentration of 80 µg/ml and were screened again on a fresh hygromycin B-containing PDA. To confirm viral RNA from FgVs-infected colonies, reverse transcriptase-polymerase chain reaction (RT-PCR) was performed followed by enzyme treatment with DNase I and S1 nuclease (Takara Bio Inc., Otsu, Japan) as described previously [2]. The genetic background of protoplast fusants was determined by amplified fragment length polymorphisms (AFLPs) and Southern blot hybridization as described previously [2]. To obtain hygromycin-sensitive colonies containing mycovirus, virus transmission was conducted using dual culture of virus-free PH-1 (wild-type; recipient) and virus-infected PH-1 (donor) on PDA. The absence of the *hygB* gene was confirmed by PCR using the primers from the *hygB* cassette. Viral RNAs were checked by RT-PCR and enzyme treatment as described above. Fungal colonies derived from anastomosis were subcultured at least three times and subjected to further analysis. Although we used hygromycin B as a selective marker, hyphal anastomosis was also examined using dual culture of virus-free and virus-infected PH-1 to avoid any adverse effects of the antibiotic resistance gene.

### Sexual development

To induce production of perithecia, 7-day-old cultures grown on carrot agar medium were treated with 1 ml of 2.5% (v/v) sterilized Tween-60 solution and then pressed down with a sterile glass spreader as previously described with minor modifications [20]. All cultures were then incubated under UV light (365 nm; HKiv Import & Export Co. Ltd., Xiamen, China) at 25°C for 7 days and observed with the SteREP Lumar V12 and AxioCam fluorescent stereoscopic microscope system (Carl Zeiss, Oberkochen, Germany).

### Conidiation and vertical transmission

For conidiation of virus-free and FgVs-infected strains, five mycelia plugs of each strain were incubated in 50 ml of carboxymethyl cellulose (CMC) medium (1.5% carboxymethyl cellulose, 0.1% yeast extract, 0.05% MgSO_4_•7H_2_O, 0.1% NH_4_NO_3_, and 0.1% KH_2_PO_4_) at 25°C and 150 rpm for 5 days. Conidia produced in CMC culture were filtered through six layers of sterilized gauze, collected by centrifugation, and counted. Virus transmission was measured by allowing cultures to sporulate, performing 100 conidia isolation per strain, and assessing each conidium for the specific mycovirus. Agar plugs from virus-positive cultures were used to start the next generation, and three generations of conidia were generated and assessed. The presence of viral RNA from the FgVs-positive single conidial isolates was determined by RT-PCR using specific primers and enzyme treatment with DNase I and S1 nuclease (Takara Bio Inc).

### Virulence assays

Virulence was assayed as previously described [20]. A conidial suspension of each strain was injected into 15 replicate wheat head florets at early-mid anthesis. Virulence was assessed 14 days after inoculation by determining the percentage of spikelets with head blight symptoms. The experiment was conducted twice. Statistical analysis was performed with the PASW statistics software 20.0 (IBM SPSS Inc., Armonk, U.S.A.).

### RNA extraction and dsRNA purification

Frozen mycelia were ground to a fine powder in liquid nitrogen and with a mortar and pestle. Total RNAs were extracted with Iso-RNA Lysis reagent (5 PRIME, Gaithersburg, USA) according to the manufacturer's instructions, followed by treatment with DNase I (Takara Bio Inc) to remove genomic DNA completely. The samples were extracted with phenol-chloroform, precipitated with ethanol, and finally suspended in DEPC-treated water. The dsRNAs from total RNAs of fungal strains were purified through a Whatman CF11 cellulose column (GE Healthcare), separated on a 5% polyacrylaminde gel, and visualized on a UV transilluminator after ethidium bromide straining.

### Trichothecene analysis

For mycotoxin analysis, conidia of virus-free and virus-infected strains were harvested in 50 ml of CMC culture at days after inoculation, as described previously [18]. Conidial suspensions (2×10^5^ conidia per dish) were grown in 20 ml of defined media containing 5 mM agmatine. Three replicates of each strain were used for this experiment. Mycotoxin was extracted from the filtrates and analyzed with a Shimadzu QP-5000 gas chromatograph–mass spectrometer as described previously [18]. The trichothecenes were measured based on the biomass produced by each strain.

### Preparation of cDNA library and sequencing

The cDNA library for sequencing was constructed using Illumina TruSeq mRNA Sample Prep Kit v2 according to the manufacturer's instruction. In brief, poly(A) tailed mRNAs were isolated by oligo(dT) selection using Dynabeads magnetic beads (Invitrogen, Carlsbad, U.S.A.). The isolated mRNAs were randomly fragmented by Mg^2+^ ions, and then the double-stranded cDNA was synthesized by SuperScript II Reverse Transcriptase (Invitrogen). After cDNAs were repaired, adaptors were ligated to the ends of the cDNA fragments. PCR was performed to enrich the purified cDNA template with approximately 200-bp fragments. The quality of the constructed cDNA template was assessed with an Agilent Technologies 2100 Bioanalyzer using the Agilent DNA 1000 chip kit (Agilent, Santa Clara, U.S.A.). Sequencing was performed using the Illumina HiSeq2000 (Illumina, San Diego, U.S.A.) at the National Instrumentation Center for Environmental Management (NICEM) of the Seoul National University. The raw sequencing data are available from the NCBI Sequence Read Archive (SRA) under accession numbers: SRR1185280-SRR1185283, SRR1185285.

### RNA-Seq analysis

All sequenced libraries were subjected for mapping on the reference genome of *Fusarium graminearum* PH-1 derived from (http://www.broadinstitute.org/) using TopHat program [29]. Expression values were obtained by calculating FPKM (Fragments Per Kilobase of transcript per Million mapped reads). Differentially gene expression was analyzed by calculating fold changes and several statistical tests using Cufflinks program [29]. DEG were identified based on more than two-fold changes and p-value less than 0.05.

### Gene ontology enrichment analysis

Because of the poor annotation for *Fusarium* genes, we first annotated all 13,321 *Fusarium graminearum* genes using the Blast2GO program [30]. In addition, gene ontology (GO) terms, enzyme codes, and InterPro domains for individual gene were obtained. GO enrichment analyses were performed using Fisher's exact test with multiple testing corrections with a false discovery rate (FDR) <0.05.

### Total RNA preparation and real time RT-PCR

For total RNA preparation, the powdered mycelia were suspended in Isol-RNA lysis reagent (5 PRIME, Hilden, Germany). Nucleic acid was extracted following the manufacturer's protocol with slight modification. The extracted total RNAs were purified twice with acid phenol:chloroform (1∶1), precipitated with isopropanol, suspended in DEPC-treated water, and further treated with TURBO DNA-free (Ambion, Austin, U.S.A.) to remove genomic DNA. The cDNAs were synthesized with M-MLV reverse transcriptase (Promega) and oligo d(T) primer to quantify mRNA expression. Quantitative real-time RT-PCR (qRT-PCR) was performed on a CFX96 Real-Time PCR System (Bio-Rad, Hercules, U.S.A.) using the SsoFast EvaGreen Supermix (Bio-Rad) according to manufacturer's instructions. After initial denaturation at 95°C for 10 min, 40 cycles consisted of 5 s at 95°C and 5 s at 58°C. Two endogenous reference genes, cyclophilin 1 (CYP1, locus FGSG_07439) and elongation factor 1α (EF1α, locus FGSG_08811), were used as reference genes to normalize real time RT-PCR results.

## Supporting Information

File S1 **Combined file of supporting figures. Figure S1. Screening of FgVs 1-4-infected PH-1 strains.** Virus-infected strains (I: FgV1, II: FgV2, III: FgV3, and IV: FgV4) obtained by fusion experiment were screened by enzyme treatment (A), RT-PCR analysis (B), Southern blot hybridization (C), and AFLP (amplified fragment length polymorphism) fingerprinting (D). Lane M, λ DNA; lane M1, λ DNA-*Hin*dIII digested DNA marker; lane M2, 1-kb ladder (Bioneer, Daejeon, Korea). (A) S1 nuclease and DNase I treatment. DK21, 98-8-60, and DK3, donor strains used in this study; lanes 1–8, protoplast fusants. (B) RT-PCR analysis of virus-infected strains. Presence of viral dsRNA was confirmed by RT-PCR amplification with a primer pair designed from the RdRp coding region of each virus. PCR products were separated on 1% agarose gel. No template, negative control; Lanes DK21, 98-8-60, and DK3, positive control; lanes 1–8, protoplast fusants. (C) Southern blot hybridization. Lane 1, PH-1 (wild-type); lane 2, hygromycin B-resistant PH-1 (positive control); lanes DK21, 98-8-60, and DK3, donor strains. Genomic DNAs extracted from protoplast fusants were digested with *Bam*HI and hybridized with PCR fragments from the hygromycin resistance B cassette of pCB1004. (D) AFLP fingerprinting. Lane 1, PH-1 (wild-type); lane 2, hygromycin B-resistant PH-1; lane 3, virus-free donor strain; lane 4, donor strain; lanes 5–11, protoplast fusants. Genomic DNAs of λ DNA and fungal strains were amplified with the primer combinations *Eco*RI+0/*Mse*I+0 and *Eco*RI+CA/*Mse*I+GC, respectively. (+0 indicates no selective nucleotides, +CA and +GC indicate selective nucleotides). The molecular weights of the fingerprints ranged from 60–440 nucleotides. **Figure S2. Alignment of histone H3 sequences from the** ***Fusarium*** **strains.** The fixed nucleotide characters are shaded in green (*F. asiaticum*; lineage 6) or yellow (*F. graminearum*; lineage 7). The presence of nucleotides G (position 278) and T (position 279) is differentially fixed for *F. asiaticum* and *F. graminearum*, respectively. The GenBank accession numbers of the nucleotide sequences that were used are as follows: NRRL 5883 (AY452815.1), NRRL 6394 (AY452817.1), NRRL 13383 (AY452819.1), NRRL 28063 (AY452816.1), NRRL 28336 (AY452818.1), NRRL 29169 (AY452836.1), and NRRL 31084 (PH-1; AY452852.1). In a previous report [2], we described the molecular identification of DK21 (*F. boothii*; lineage 3) and DK3 (*F. graminearum*; lineage 7). **Figure S3. Morphology of asci rosettes of** ***F. graminearum*** **PH-1 strains.** Each strain was grown on carrot agar for 7 days. After treatment with a Tween-60 solution, all cultures were incubated under UV light for 7 days. Scale bar  = 200 µm. **Figure S4. Conidial morphology of** ***F. graminearum*** **PH-1 strains.** Conidia harvested from 5-day-old CMC cultures were examined with a light microscope. Scale bar  = 50 µm. **Figure S5. Enriched GO terms according to biological process in the group of DEGs that were down-regulated in response to FgV4 infection.** A GO diagram of significantly over-represented GO terms (in the group of DEGs that were down-regulated by FgV4 infection) related to biological process. **Figure S6. Enriched GO terms according to cellular component in the group of DEGs that were down-regulated in response to FgV4 infection.** A GO diagram of significantly over-represented GO terms (in the group of DEGs that were down-regulated by FgV4 infection) related to cellular component. **Figure S7. Comparison between RNA-Seq and microarray data.** The numbers of DEGs were compared among three data sets obtained from FgV1 infection. RSeq 120 h indicates RNA-Seq data sampled at 120 hours post infection while Micro 36 h and Micro 120 h indicate microarray data which were obtained at 36 and 120 hours post infection, respectively.
(PDF)

File S2 **Combined file of supporting tables. Table S1.** Expression ratios (fold-change relative to the virus-free wild type) of *Fusarium graminearum* genes in each virus-infected sample. **Table S2.** The 20 genes in each virus-infected strain with the greatest difference in expression relative to that in the wild type (PH-1). **Table S3.** GO enrichment analysis of the DEGs in each sample. **Table S4.** Expression ratios (fold-change relative to the virus-free wild type) of *Fusarium graminearum* TFs in each virus-infected sample. **Table S5.** Expression ratios (fold-change relative to the virus-free wild type) of *Fusarium graminearum* genes involved in post transcriptional gene silencing. **Table S6.** List of primers used for real time RT-PCR.
(XLSX)
