# Supplementary material for: A Comparison of Transcriptional Patterns and Mycological Phenotypes following Infection of Fusarium graminearum by Four Mycoviruses
Source: PLoS One. 2014 Jun 25;9(6):e100989. doi: 10.1371/journal.pone.0100989 (PMC4071046; doi:10.1371/journal.pone.0100989)

Figure S1

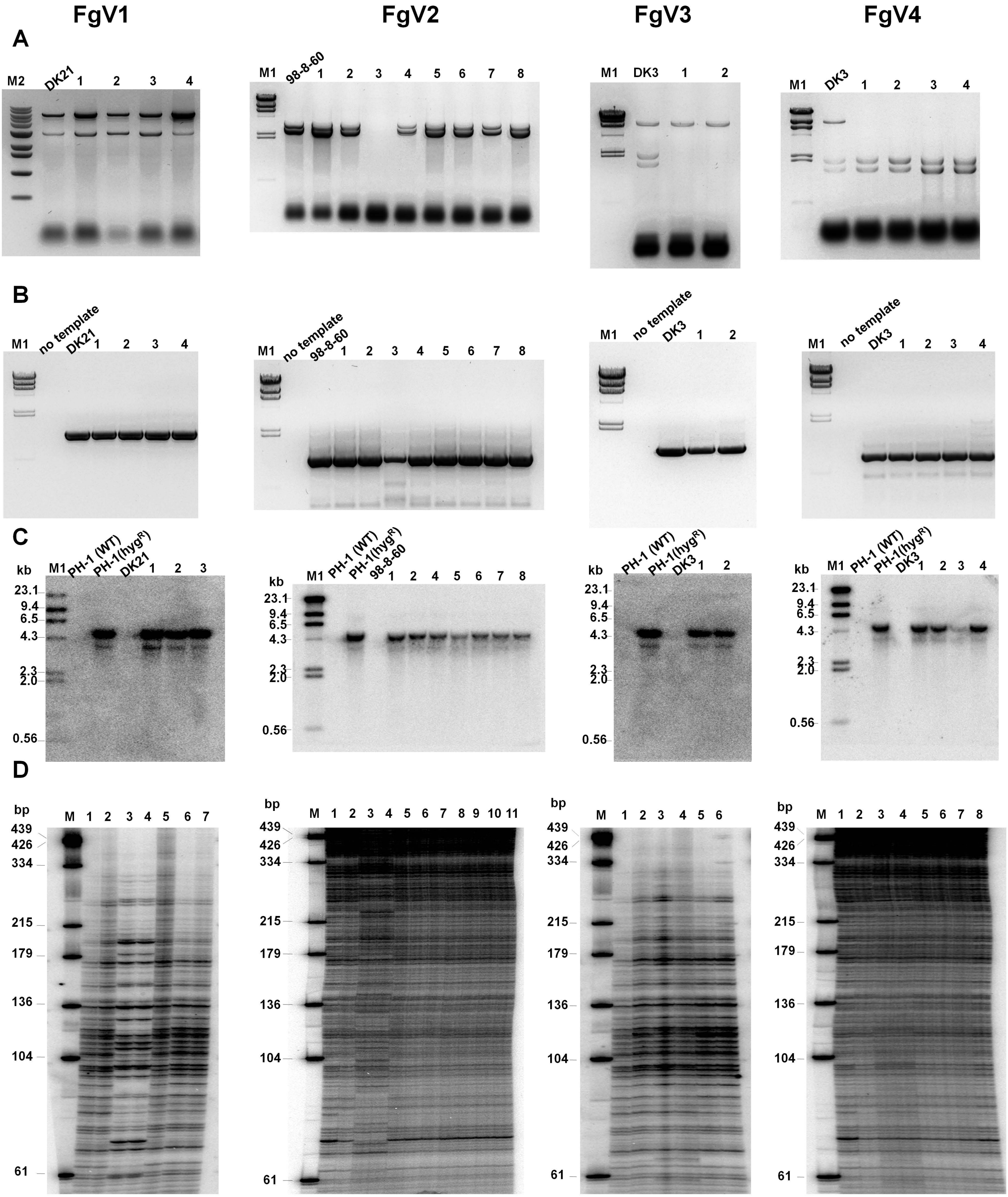

FgV1
FgV2
FgV3
FgV4
A
M2 DK21 1 2 3 4
M1 98-8-60 1 2 3 4 5 6 7 8
M1 DK3 1 2
M1 DK3 1 2 3 4
B
M1 no template DK21 1 2 3 4
M1 no template 98-8-60 1 2 3 4 5 6 7 8
M1 no template DK3 1 2
M1 no template DK3 1 2 3 4
C
kb M1 PH-1 (WT) PH-1(hyg^R) DK21 1 2 3
kb M1 PH-1 (WT) PH-1(hyg^R) 98-8-60 1 2 4 5 6 7 8
kb PH-1 (WT) PH-1(hyg^R) DK3 1 2
kb M1 PH-1 (WT) PH-1(hyg^R) DK3 1 2 3 4
23.1
9.4
6.5
4.3
2.3
2.0
0.56
D
bp M 1 2 3 4 5 6 7
bp M 1 2 3 4 5 6 7 8 9 10 11
bp M 1 2 3 4 5 6
bp M 1 2 3 4 5 6 7 8
439
426
334
215
179
136
104
61

Figure S2

Histon H3 position

***F. asiaticum*- 278 (G), *Fusarium graminearum*- 279(T)**

```
                        220         *       240         *       260         *       280
DK21        : GCTTCTCATCCGAAAGCTCCCCTTCCAGCGTCTCGTAAGCACCACCTGAACATCGCATCATCGCTTGACA
98-8-60     : GCTTCTCATCCGAAAGCTCCCCTTCCAGCGTCTCGTAAGCATCACCTGAACATCGCATCATCGCATGGCA
DK3         : GCTTCTCATCCGAAAGCTCCCCTTCCAGCGTCTCGTAAGCATCACCCGAACATCGCATCATCGCTTGATA
NRRL_5883   : GCTTCTCATCCGAAAGCTCCCCTTCCAGCGTCTCGTAAGCATCACCCGAACATCGCATCATCGCTTGATA
NRRL_6394   : GCTTCTCATCCGAAAGCTCCCCTTCCAGCGTCTCGTAAGCATCACCCGAACATCGCATCATCGCTTGATA
NRRL_13383  : GCTTCTCATCCGAAAGCTCCCCTTCCAGCGTCTCGTAAGCATCACCCGAACATCGCATCATCGCTTGATA
NRRL_28063  : GCTTCTCATCCGAAAGCTCCCCTTCCAGCGTCTCGTAAGCATCACCCGAACATCGCATCATCGCTTGATA
NRRL_28336  : GCTTCTCATCCGAAAGCTCCCCTTCCAGCGTCTCGTAAGCATCACCCGAACATCGCATCATCGCTTGATA
NRRL_29169  : GCTTCTCATCCGAAAGCTCCCCTTCCAGCGTCTCGTAAGCATCACCCGAACATCGCATCATCGCTTGATA
NRRL_31084  : GCTTCTCATCCGAAAGCTCCCCTTCCAGCGTCTCGTAAGCATCACCCGAACATCGCATCATCGCTTGATA
PH-1(FgV1)  : GCTTCTCATCCGAAAGCTCCCCTTCCAGCGTCTCGTAAGCATCACCCGAACATCGCATCATCGCTTGATA
PH-1(FgV2)  : GCTTCTCATCCGAAAGCTCCCCTTCCAGCGTCTCGTAAGCATCACCCGAACATCGCATCATCGCTTGATA
PH-1(FgV3)  : GCTTCTCATCCGAAAGCTCCCCTTCCAGCGTCTCGTAAGCATCACCCGAACATCGCATCATCGCTTGATA
PH-1(FgV4)  : GCTTCTCATCCGAAAGCTCCCCTTCCAGCGTCTCGTAAGCATCACCCGAACATCGCATCATCGCTTGATA
              GCTTCTCATCCGAAAGCTCCCCTTCCAGCGTCTCGTAAGCAtCACCcGAACATCGCATCATCGCtTGatA
```

Figure S3

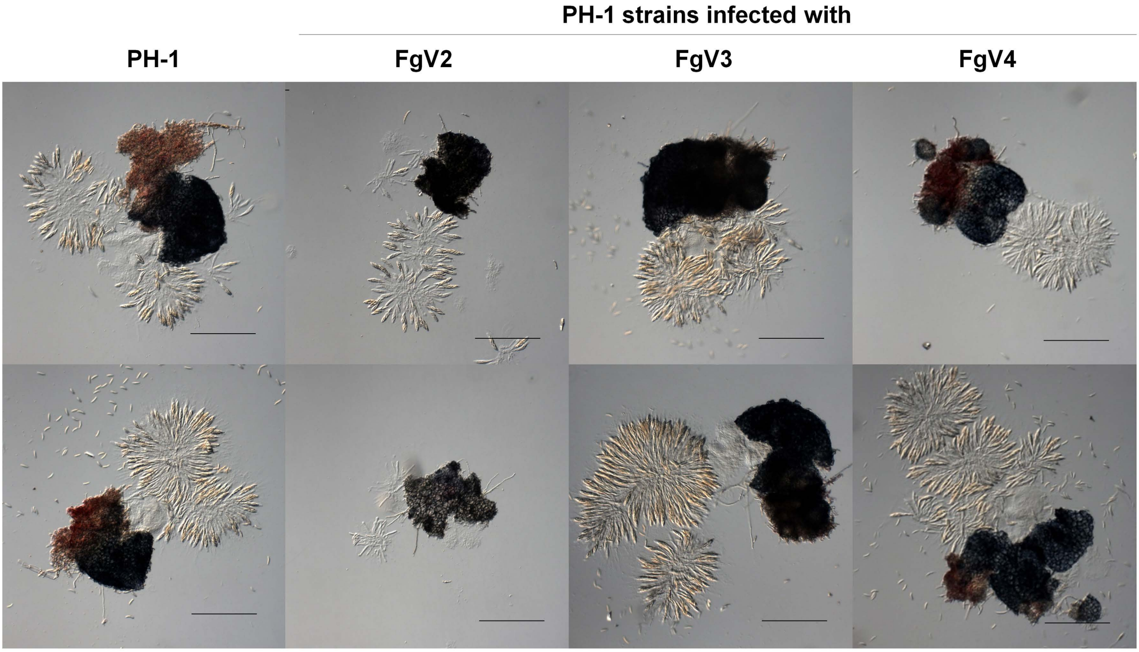

Figure S4

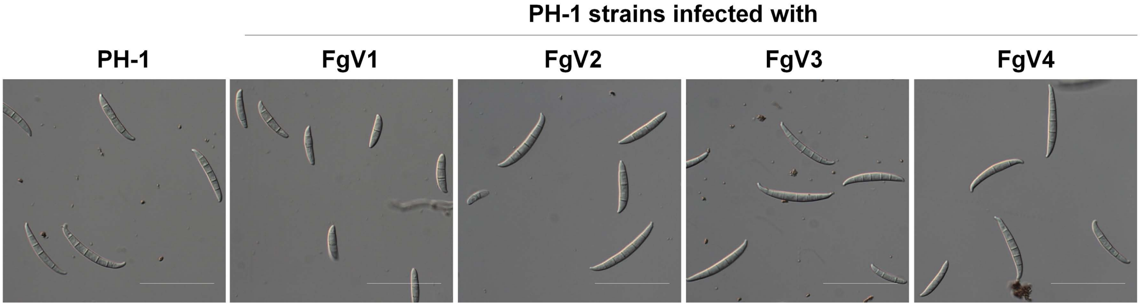

Figure S5

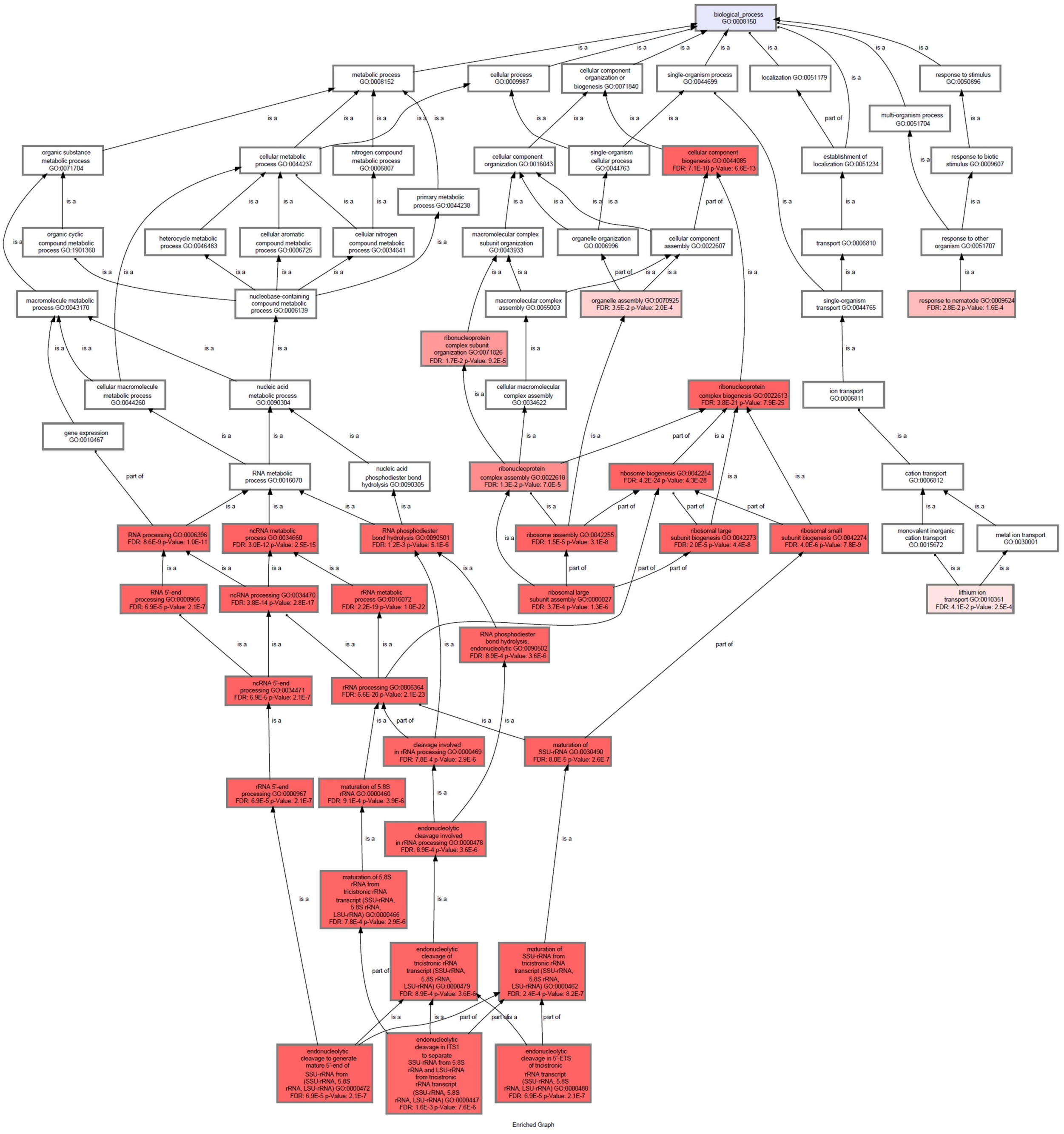

biological_process GO:0008150
metabolic process GO:0008152
cellular process GO:0009987
cellular component organization or biogenesis GO:0071840
single-organism process GO:0044699
localization GO:0051179
response to stimulus GO:0050896
multi-organism process GO:0051704
organic substance metabolic process GO:0071704
cellular metabolic process GO:0044237
nitrogen compound metabolic process GO:0006807
primary metabolic process GO:0044238
cellular component organization GO:0016043
single-organism cellular process GO:0044763
cellular component biogenesis GO:0044085 FDR: 7.1E-10 p-Value: 6.6E-13
establishment of localization GO:0051234
response to biotic stimulus GO:0009607
organic cyclic compound metabolic process GO:1901360
heterocycle metabolic process GO:0046483
cellular aromatic compound metabolic process GO:0006725
cellular nitrogen compound metabolic process GO:0034641
macromolecular complex subunit organization GO:0043933
organelle organization GO:0006996
cellular component assembly GO:0022607
transport GO:0006810
response to other organism GO:0051707
macromolecule metabolic process GO:0043170
nucleobase-containing compound metabolic process GO:0006139
macromolecular complex assembly GO:0065003
organelle assembly GO:0070925 FDR: 3.5E-2 p-Value: 2.0E-4
single-organism transport GO:0044765
response to nematode GO:0009624 FDR: 2.8E-2 p-Value: 1.6E-4
ribonucleoprotein complex subunit organization GO:0071826 FDR: 1.7E-2 p-Value: 9.2E-5
cellular macromolecule metabolic process GO:0044260
nucleic acid metabolic process GO:0090304
cellular macromolecular complex assembly GO:0034622
ribonucleoprotein complex biogenesis GO:0022613 FDR: 3.8E-21 p-Value: 7.9E-25
ion transport GO:0006811
gene expression GO:0010467
RNA metabolic process GO:0016070
nucleic acid phosphodiester bond hydrolysis GO:0090305
ribonucleoprotein complex assembly GO:0022618 FDR: 1.3E-2 p-Value: 7.0E-5
ribosome biogenesis GO:0042254 FDR: 4.2E-24 p-Value: 4.3E-28
cation transport GO:0006812
RNA processing GO:0006396 FDR: 8.6E-9 p-Value: 1.0E-11
ncRNA metabolic process GO:0034660 FDR: 3.0E-12 p-Value: 2.5E-15
RNA phosphodiester bond hydrolysis GO:0090501 FDR: 1.2E-3 p-Value: 5.1E-6
ribosome assembly GO:0042255 FDR: 1.5E-5 p-Value: 3.1E-8
ribosomal large subunit biogenesis GO:0042273 FDR: 2.0E-5 p-Value: 4.4E-8
ribosomal small subunit biogenesis GO:0042274 FDR: 4.0E-6 p-Value: 7.8E-9
monovalent inorganic cation transport GO:0015672
metal ion transport GO:0030001
RNA 5'-end processing GO:0000966 FDR: 6.9E-5 p-Value: 2.1E-7
ncRNA processing GO:0034470 FDR: 3.8E-14 p-Value: 2.8E-17
rRNA metabolic process GO:0016072 FDR: 2.2E-19 p-Value: 1.0E-22
ribosomal large subunit assembly GO:0000027 FDR: 3.7E-4 p-Value: 1.3E-6
lithium ion transport GO:0010351 FDR: 4.1E-2 p-Value: 2.5E-4
RNA phosphodiester bond hydrolysis, endonucleolytic GO:0090502 FDR: 8.9E-4 p-Value: 3.6E-6
ncRNA 5'-end processing GO:0034471 FDR: 6.9E-5 p-Value: 2.1E-7
rRNA processing GO:0006364 FDR: 6.6E-20 p-Value: 2.1E-23
cleavage involved in rRNA processing GO:0000469 FDR: 7.8E-4 p-Value: 2.9E-6
maturation of SSU-rRNA GO:0030490 FDR: 8.0E-5 p-Value: 2.6E-7
rRNA 5'-end processing GO:0000967 FDR: 6.9E-5 p-Value: 2.1E-7
maturation of 5.8S rRNA GO:0000460 FDR: 9.1E-4 p-Value: 3.9E-6
endonucleolytic cleavage involved in rRNA processing GO:0000478 FDR: 8.9E-4 p-Value: 3.6E-6
maturation of 5.8S rRNA from tricistronic rRNA transcript (SSU-rRNA, 5.8S rRNA, LSU-rRNA) GO:0000466 FDR: 7.8E-4 p-Value: 2.9E-6
endonucleolytic cleavage of tricistronic rRNA transcript (SSU-rRNA, 5.8S rRNA, LSU-rRNA) GO:0000479 FDR: 8.9E-4 p-Value: 3.6E-6
maturation of SSU-rRNA from tricistronic rRNA transcript (SSU-rRNA, 5.8S rRNA, LSU-rRNA) GO:0000462 FDR: 2.4E-4 p-Value: 8.2E-7
endonucleolytic cleavage to generate mature 5'-end of SSU-rRNA from (SSU-rRNA, 5.8S rRNA, LSU-rRNA) GO:0000472 FDR: 6.9E-5 p-Value: 2.1E-7
endonucleolytic cleavage in ITS1 to separate SSU-rRNA from 5.8S rRNA and LSU-rRNA from tricistronic rRNA transcript (SSU-rRNA, 5.8S rRNA, LSU-rRNA) GO:0000447 FDR: 1.6E-3 p-Value: 7.6E-6
endonucleolytic cleavage in 5'-ETS of tricistronic rRNA transcript (SSU-rRNA, 5.8S rRNA, LSU-rRNA) GO:0000480 FDR: 6.9E-5 p-Value: 2.1E-7
is a
part of
Enriched Graph

Figure S6

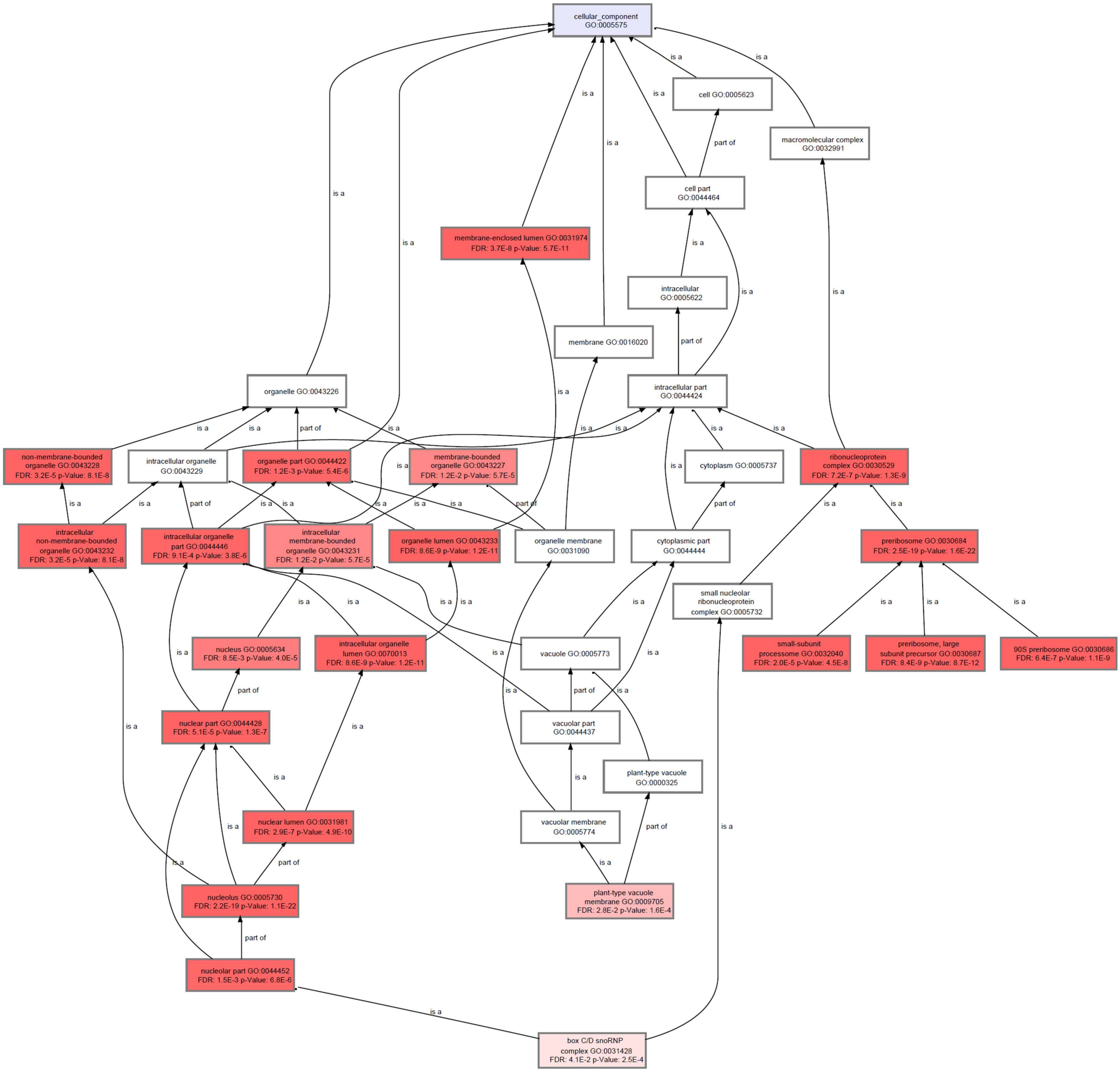

Enriched Graph

Figure S7

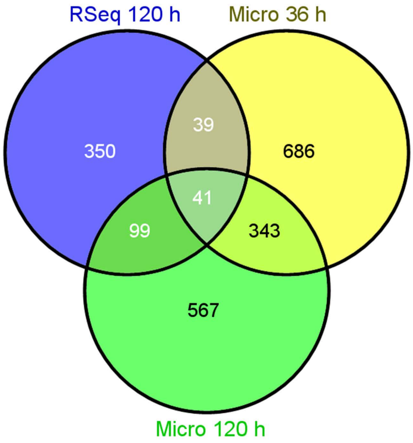

Supplement: File S1 — Combined file of supporting figures. Figure S1. Screening of FgVs 1-4-infected PH-1 strains. Virus-infected strains (I: FgV1, II: FgV2, III: FgV3, and IV: FgV4) obtained by fusion experiment were screened by enzyme treatment (A), RT-PCR analysis (B), Southern blot hybridization (C), and AFLP (amplified fragment length polymorphism) fingerprinting (D). Lane M, λ DNA; lane M1, λ DNA-HindIII digested DNA marker; lane M2, 1-kb ladder (Bioneer, Daejeon, Korea). (A) S1 nuclease and DNase I treatment. DK21, 98-8-60, and DK3, donor strains used in this study; lanes 1–8, protoplast fusants. (B) RT-PCR analysis of virus-infected strains. Presence of viral dsRNA was confirmed by RT-PCR amplification with a primer pair designed from the RdRp coding region of each virus. PCR products were separated on 1% agarose gel. No template, negative control; Lanes DK21, 98-8-60, and DK3, positive control; lanes 1–8, protoplast fusants. (C) Southern blot hybridization. Lane 1, PH-1 (wild-type); lane 2, hygromycin B-resistant PH-1 (positive control); lanes DK21, 98-8-60, and DK3, donor strains. Genomic DNAs extracted from protoplast fusants were digested with BamHI and hybridized with PCR fragments from the hygromycin resistance B cassette of pCB1004. (D) AFLP fingerprinting. Lane 1, PH-1 (wild-type); lane 2, hygromycin B-resistant PH-1; lane 3, virus-free donor strain; lane 4, donor strain; lanes 5–11, protoplast fusants. Genomic DNAs of λ DNA and fungal strains were amplified with the primer combinations EcoRI+0/MseI+0 and EcoRI+CA/MseI+GC, respectively. (+0 indicates no selective nucleotides, +CA and +GC indicate selective nucleotides). The molecular weights of the fingerprints ranged from 60–440 nucleotides. Figure S2. Alignment of histone H3 sequences from the Fusarium strains. The fixed nucleotide characters are shaded in green (F. asiaticum; lineage 6) or yellow (F. graminearum; lineage 7). The presence of nucleotides G (position 278) and T (position 279) is differentially fixed for F [file pone.0100989.s001.pdf]
